# Muon–Nitrogen Quadrupolar Level Crossing Resonance in a Charge Transfer Salt

**DOI:** 10.1021/acs.jpcc.2c00617

**Published:** 2022-04-26

**Authors:** Adam Berlie, Francis L. Pratt, Benjamin M. Huddart, Tom Lancaster, Stephen P. Cottrell

**Affiliations:** † ISIS Neutron and Muon Source, STFC Rutherford Appleton Laboratory, Chilton, Oxfordshire OX11 0QX, United Kingdom; ‡ Department of Physics, Durham University, South Road, Durham DH1 3LE, United Kingdom

## Abstract

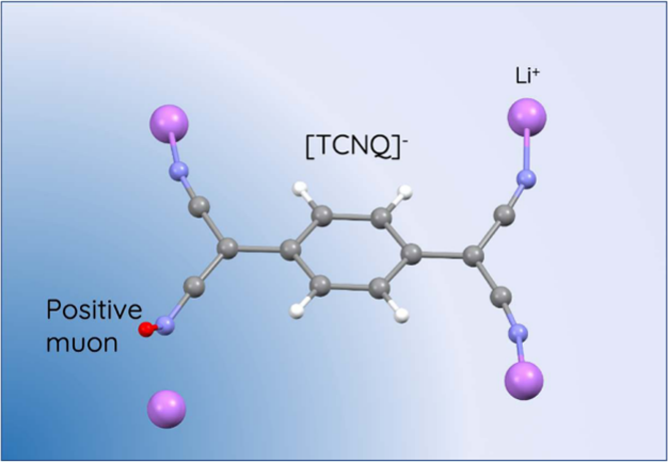

Although muons are primarily regarded as a local spin probe, they can also access the charge state of an atom or molecule via quadrupolar level crossing resonance (QLCR) spectroscopy. We use Li^+^TCNQ^–^ (TCNQ = 7,7,8,8-tetracyanoquinodimethane), a simple charge transfer salt, to test the potential of this technique in molecular systems by studying the interaction of a positive muon with the TCNQ nitrogen atoms. We show that both a positive muon and muonium are able to add to the nitrogen, leading to a singlet spin state for the addition molecule. This produces a characteristic three line QLCR spectrum, with the observed line positions and intensities determined by the principal values and orientation of the electric field gradient tensor at the nitrogen. Ab initio calculation of this field gradient and the resulting QLCR spectrum give good agreement with the experiment. A nonresonant background contribution to the relaxation rate also provides evidence for spin excitations rapidly diffusing along the TCNQ chains. These reflect mobile unpaired electrons introduced by muonium addition. It is thus shown that a single set of muon measurements can be sensitive to both spin and charge degrees of freedom in the same molecular material.

## Introduction

Muon spin spectroscopy (also known as muon spin relaxation/rotation/resonance, μSR) is a valuable probe that is able to provide detail on both the local and bulk properties of materials.^1−4^ The common convention is to use the positive muon (anti-muon, μ^+^ and will henceforth be referred to as the “muon”), which is analogous to a proton but with 1/9th of the mass. The muon is implanted, generally with MeV energies, and after a series of ballistic and inelastic scattering processes thermalizes at a specific site within the chemical structure. The muon will then generally be static within the chemical structure; however, there are also cases where the muon is able to hop between sites. Information about the sample is gained from the time evolution of the muon spin, measured over many lifetimes of the muon (average life of the muon is 2.2 μs), due to coupling to the local magnetic field distribution. The simplest state that the muon can form is one where the bare muon sits within an interstitial site^5^ or forms a sigma bond with a lone pair on an atom, for example, the lone pair on an oxygen ion within an oxide structure.^6^ This is known as a diamagnetic state. More complex final states involve the muon capturing an electron from atoms or molecules as it thermalizes, forming muonium (μ^+^e^–^), analogous to hydrogen.^7,8^ The muonium can then undergo a chemical addition to a molecule. For example, within benzene, the muonium adds across a C=C double bond to form the cyclohexadienyl radical,^1,9−11^ which is known as a paramagnetic state. Therefore, there is interest in how the muon interacts with materials on an atomic and molecular level to help understand the broad range of data that are collected to describe the physical properties of compounds.

**Figure 1:**
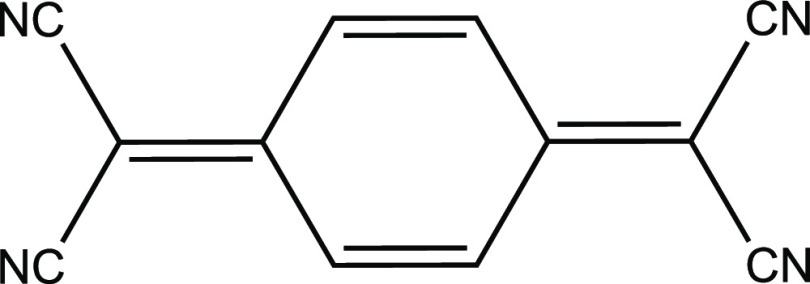
Molecular structure of TCNQ.

One area where μSR experiments can provide some unique insight is in the field of magnetism and magnetic materials, in particular, when one is studying materials based on organic molecules.^2,12,13^ The relatively weak and delocalized electronic magnetic moment on the organic molecule can prove difficult to measure using other techniques. Often, these organic-based materials take the form of charge transfer compounds^14,15^ and they can also display other interesting physical properties such as electrical conductivity,^16^ ferroelectricity, and multiferroic behavior.^17,18^ A prominent organic molecule used within these types of compounds is TCNQ (7,7,8,8-tetracyanoquinodimethane), a planar-conjugated molecule that is capable of stabilizing a radical anion leading to an *S* = 1/2 state, as shown in Figure 1.

For neutral TCNQ, it is well known that there is muonium addition to one of the nitrogens, resulting in the formation of a paramagnetic radical.^19^ Using Avoid Level Crossing (ALC) resonance, one is able to study the muon-radical hyperfine interaction, and in this case, the contact hyperfine coupling constant is found to be 85 MHz.^19^ Muonium addition and the formation of a paramagnetic state are also accompanied by a fast muon spin depolarization. Depending on the muon source used,^4,20^ this depolarization can either be observed directly or present as the loss of signal amplitude when the muon spin is dephased before the start of the experimental time scale. Interestingly, for TCNQ-based charge transfer salts, when one already starts with an *S* = 1/2 radical using the reduced form of TCNQ, there is no missing asymmetry, and no paramagnetic state is formed.^21−27^ In this case, it is important to understand how the muon interacts with the molecule and determine what information one can gain from this interaction, with the results being applicable to other TCNQ-based charge-transfer solids.

In this study, we use the TCNQ charge transfer compound, Li^+^TCNQ^–^, that is, a 1:1 stoichiometric salt having a stacked molecular chain structure, which is expected to form a spin-Peierls dimerized ground state at ∼220 K.^28^ In order to understand how the muon interacts with the sample, we investigate the muon coupling to the quadrupolar moment on the ^14^N nuclei and study the resonance of the muon-nuclear quadrupole coupling, known as muon quadrupole level crossing resonance (μ-QLCR or simply QLCR). The resulting spectrum is compared with the results of calculations and we are able to show how the muon bonds to the TCNQ molecule, determining the orientation between the muon bond and the C–N bond. This provides us with a precise characterization of the muon stopping site within this organic charge-transfer material, determining the coupling of the local muon probe to both spin and charge fluctuations within the system. The method should be generally applicable to other molecular materials in which a muon site is in close proximity to a quadrupolar nucleus.

## Experimental Details

Lithium TCNQ was synthesized using the method outlined by Melby et al.^29^ Muon spin spectroscopy experiments were performed on the HiFi spectrometer at the ISIS Neutron and Muon Source. The muon data set is available from the ISIS facility.^30^ Within the experiment, 100% spin polarized muons are implanted into the sample, with the initial polarization along the direction of the beam (*z* direction). The muon spin is then dephased by the surrounding nuclear and electronic fields; QLCR experiments were conducted by sweeping a longitudinal field that is along the *z*-direction. When the Zeeman splitting of the muon matches a quadrupolar nuclear energy level splitting, a cross resonance occurs and there is a corresponding flipping of the muon polarization.^31^ Calculations of the local structure around the muon and the electric field gradient (EFG) at the nitrogen were performed using plane-wave density functional theory (DFT) using the CASTEP code^32^ and the PBE/GGA approximation.^33^ Simulation of QLCR spectra was made using the CalcALC program.^34^

## Experimental Results

**Figure 2:**
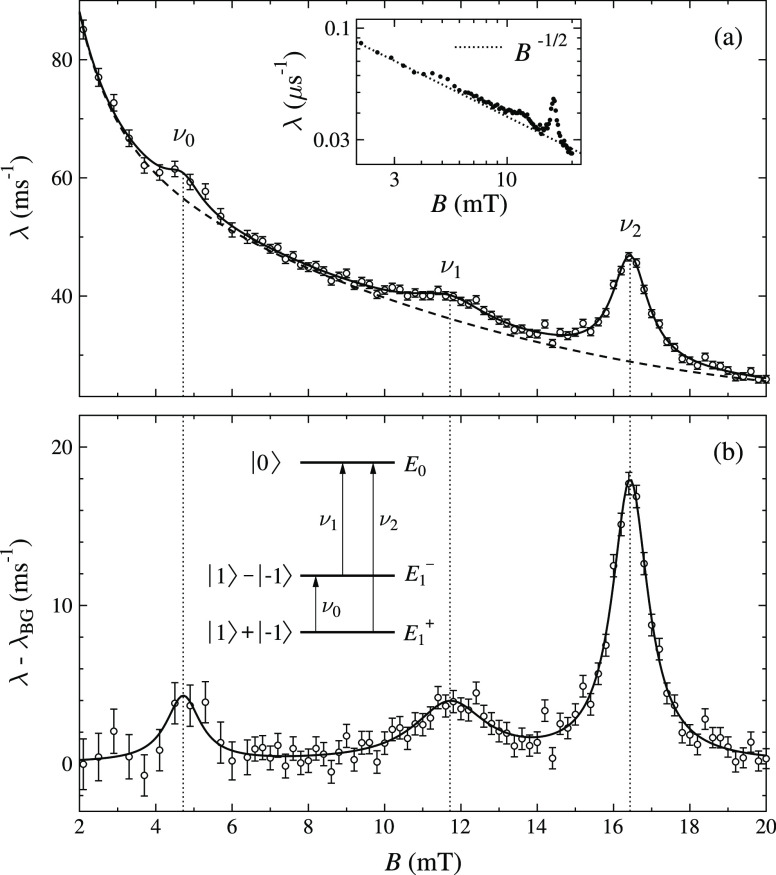
(a) Muon spin relaxation rate for Li^+^TCNQ^–^ measured at 90 K as a function of field, and the dashed line is the background modeled by a quadratic order polynomial. The inset shows that the background is also well represented by a *B*^–1/2^ power law, demonstrating the presence of 1D spin diffusion (see discussion). (b) Subtraction of the background shows clearly the three peaks that correspond to resonances with the nitrogen nuclear quadrupole energy level splittings shown in the inset. The fitted values are *C*_*Q*_ = −2.51(1) MHz and η = 0.50(1) (the negative sign of *C*_*Q*_ is inferred from a DFT calculation of the muon site geometry).

The time spectra of forward–backward muon decay positron asymmetry were measured as a function of field *B* and the best overall fit to the data was obtained using an exponential relaxation function
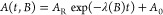
1where *A*_R_ is the relaxing asymmetry, λ(*B*) is the field-dependent muon spin relaxation rate, and *A*_0_ is a baseline nonrelaxing asymmetry component. The application of a magnetic field causes Zeeman splitting of the muon energy levels. When the muon Zeeman splitting of the energy levels equals an energy level separation of the ^14^N, this leads to successive resonances at the particular fields where the muon Zeeman splitting matches the ^14^N nuclear quadrupolar splittings. This is illustrated in the inset of Figure 2b, showing the transitions between different energy levels resulting in different frequencies, denoted as ν_*n*_ (*n* = 0, 1, 2). The relaxation rate reflects the dynamics of the muon spin and so, at resonance, where there is a slow, relative to the experimental time scale, exchange of spin polarization between the muon and the quadrupolar levels of the nitrogen, this will be observed as an apparent increase in the relaxation rate. The presence of the QLCR resonance is indicative of the final stopping site of the muon being diamagnetic, as paramagnetic states tend to be dominated by the muon–radial interactions. Figure 2a shows λ as a function of the field at 90 K where there is a clear evidence for three resonance peaks. These are superimposed on a smooth background that is modeled by a polynomial function. After removing this background, the peaks in λ are clearly observable, as shown in Figure 2b. Each peak shows a quadrupole resonance transition reflecting a quadrupolar level splitting frequency (ν_i_). The resonance fields are given by *B*_res_ = 2πν_i_/γ_μ_, where γ_μ_ is the gyromagnetic ratio of the muon. The inset in Figure 2b shows the three transitions responsible for the observed resonances. The resonance peaks were clearly observable at low temperatures; however at 298 K, the intensity was extremely weak. This loss of intensity is expected because at high temperatures, there is a significant population of low energy vibrational modes^35^ that cause distortions of the TCNQ molecule and these will be expected to smear out the interactions between the muon and the ^14^N nuclei.

### Determination of the EFG

In the low field limit that applies here, the energy levels of a quadrupolar nucleus are determined by the zero-field quadrupolar splitting and are independent of field and orientation. The quadrupolar Hamiltonian for nuclear spin *I* is given by
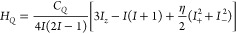
2where the nuclear quadrupole coupling constant *C*_*Q*_ = e^2^q*Q*, with eq being the principal value of the EFG and *Q* the quadrupole moment of the nucleus, where for nitrogen, we have *Q* = 2.044 fm^2^. The parameter η reflects the anisotropy of the EFG tensor *V* via η = (*V*_*xx*_ – *V*_*yy*_)/*V*_*zz*_ where *V*_*zz*_ = eq is the principal value. When *V* is axial, one has η = 0, where only one transition is observable,^36^ but in general, η can vary between 0 and 1. In the case of the N nucleus, we have *I* = 1 and the energy levels can be written as
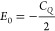
3
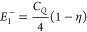
4
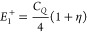
5

The *E*_0_ level corresponds to the *m*_I_ = 0 state and *E*_1_^±^ correspond to symmetric and antisymmetric combinations of the *m*_I_ = −1 and *m*_I_ = +1 states (see inset in Figure 2b). The three transitions between these levels are then given by
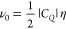
6
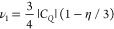
7
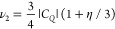
8noting that ν_0_ = ν_2_ – ν_1_. The value of |*C*_*Q*_| can be determined from the average of ν_1_ and ν_2_ and the value for η can then be determined from the splitting between ν_2_ and ν_1_, that is
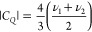
9
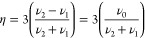
10

The λ(*B*) data of Figure 2 were fitted to the sum of three Lorentzian peaks with positions determined by *C*_*Q*_ and η as fit parameters. The amplitudes and widths for the peaks were additional free parameters in the fit, along with parameters defining the background. From this free fit, the EFG parameters were obtained directly as |*C*_*Q*_| = 2.51(1) MHz and η = 0.50(1), and so, the EFG in this case is not purely axial. Note that the sign of *C*_*Q*_ can not be determined directly from the observed transitions, but a negative sign is indicated by our DFT calculations of the EFG, which is covered in the following section.

**Figure 3:**
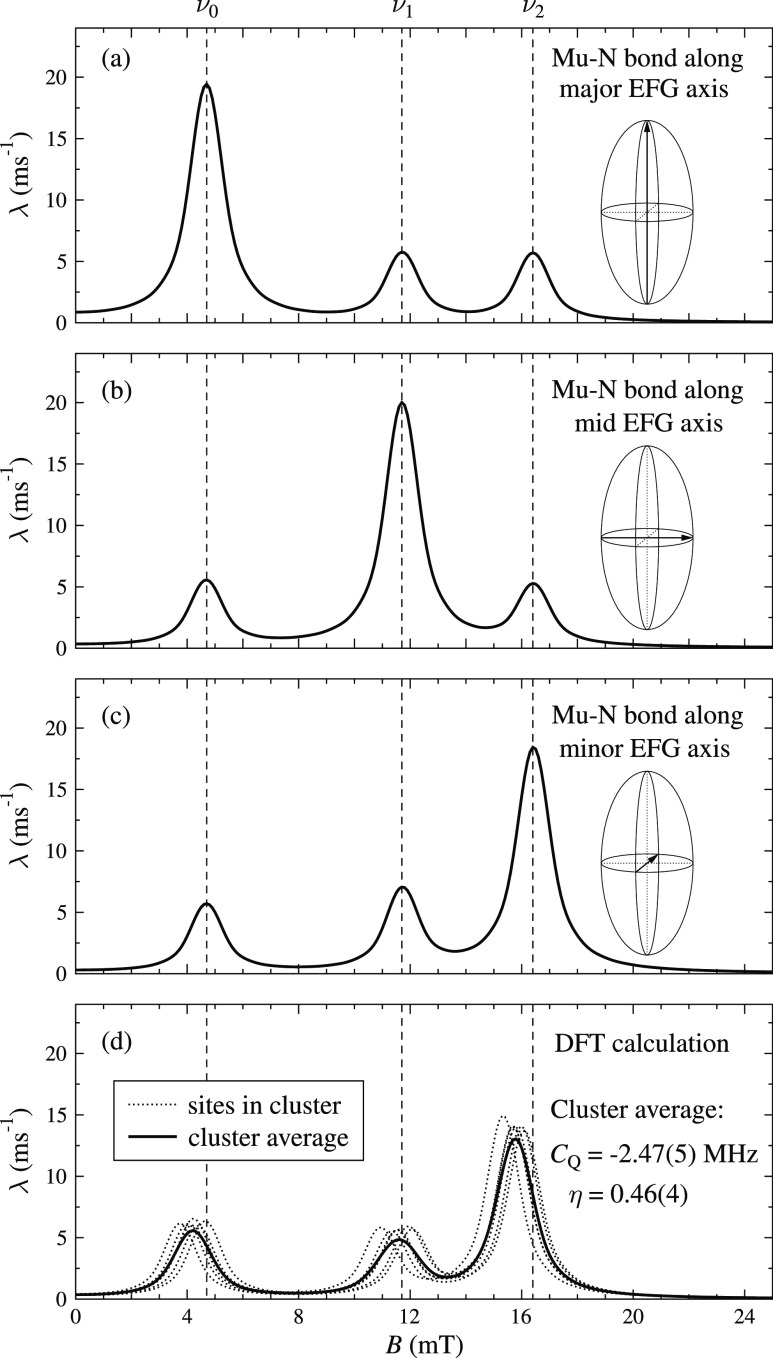
(a–c) Calculated intensities of the resonance peaks for different orientations of the EFG tensor with respect to the orientation of the muon–nitrogen bond (illustrated graphically in each case). The calculation is for a polycrystalline average using the fitted values of *C*_*Q*_ and η. Comparison with the observed ratio of intensities in Figure 2 clearly indicates alignment with the minor EFG axis. (d) Resonance peaks corresponding to the EFG tensor calculated using DFT at six similar but distinct muon sites associated with addition to the nitrogen. The average of the calculated *C*_*Q*_ and η values in the cluster and the corresponding peak intensity ratios in the spectrum all match well with the experiment.

While the principal values of the EFG tensor have been obtained from the resonance positions, further information about the local orientation of the EFG tensor can be obtained from the relative intensities of the three transitions. The principle is illustrated in Figure 3a–c where the strength of the field-dependent resonances has been derived using CalcALC with the fitted EFG parameters and varying orientation of the principal axis frame. The strongest transition as shown in Figure 3a–c depends on the orientation of the dipolar coupling axis between muon and N (i.e., the Mu–N bond) with respect to the principal axes of the EFG. From comparison of Figure 3a–c and Figure 2, it can be seen clearly that the Mu–N bond is oriented most closely with the minor axis of the EFG (the minor axis is defined here as the one whose principal value has the smallest magnitude). Figure 3d shows the resonances expected from DFT for the addition of a muon to the nitrogen on the TCNQ molecule, shown for six similar relaxed sites, which all have the EFG along the minor axis (see the next section for details of the calculations and the Supporting Information for the structures and their properties). The average properties of this cluster are *C*_*Q*_ = −2.47(5) MHz and η = 0.46(4), which provide a good match to the measured resonance peaks. Because from the general molecular geometry of TCNQ, we expect the major axis to be close to the CN axis, the intermediate axis in the plane of the molecule, and the minor axis perpendicular to the molecule; the experimental data indicate that the Mu–N bond is closest to the perpendicular direction. These QLCR measurements and associated calculations (Figure 3a–c) have thus allowed the general character of the final state of the muon to be deduced, even before using ab initio DFT to make more detailed calculations of the relaxed structure of the muon site (Figure 3d). Such DFT calculations are the subject of the next section.

### Muon State Calculations Using DFT

In recent years, there has been a rapid development of the use of DFT techniques for predicting and interpreting the properties of implanted muons in materials.^4,37^ This approach, sometimes called DFT+μ, allows for relaxed site geometry and local distortion to be determined. Electronic properties such as the hyperfine coupling and EFG can also be computed.

Because a published crystal structure is not available for Li^+^TCNQ^–^, we took the low-temperature structure of K^+^TCNQ^–^ as a starting point for the analysis. This K^+^TCNQ^–^ structure was confirmed to be stable with plane wave DFT using the PBE functional and ultrasoft pseudopotentials, as provided by the CASTEP code. In this structure, each K^+^ ion is coordinated to eight nitrogen atoms on eight different TCNQ molecules at a distance of 3 Å and each nitrogen is coordinated to two K^+^ ions. On replacing K^+^ with the significantly smaller Li^+^ ion and relaxing the structure, each nitrogen becomes coordinated to a single Li^+^ ion at the closer distance of 2 Å and each Li^+^ ion coordinates to four nitrogens rather than the eightfold coordination found in the K^+^TCNQ^–^ structure. In this case, Van der Waals dispersion force corrections were needed to stabilize the structure.

**Figure 4:**
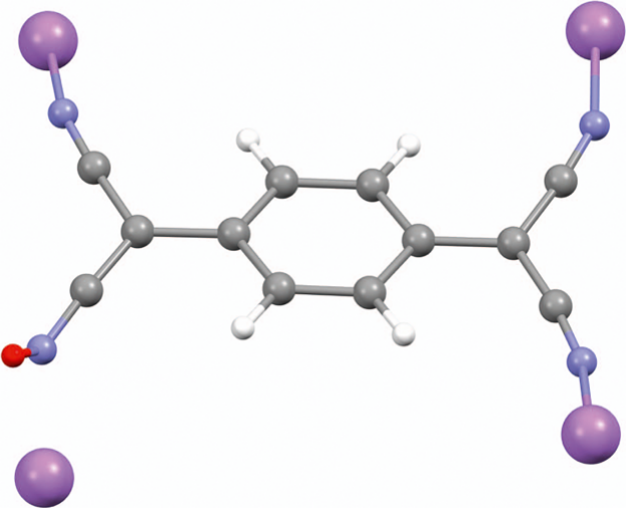
Geometry of the most stable state for Mu^+^ addition to Li^+^TCNQ^–^ calculated using DFT. The muon is shown in red, the N atoms in blue, and the Li atoms in purple. The addition of the muon has broken the bond of the N to the Li shown at the bottom left of the figure. The computed parameters for this site are −2.37 MHz for *C*_*Q*_ and 0.50 for η. The minor axis of the EFG tensor is found to be most closely aligned with the Mu–N bond axis, making an angle of 25° to it. The geometry and electronic parameters of the calculated state are reasonably consistent with the measured QLCR spectrum. A cluster of six similar sites were found in the full set of DFT calculations.

Taking this relaxed structure as the starting point, muons were introduced at fifty trial sites that were chosen randomly over the reduced cell of the stable structure by the MuFinder program,^38^ such that each site was at least 1Å away from any atom and at least 0.5 Å away from any other trial site. These trial structures were relaxed and the six lowest energy sites represent the muon addition to the nitrogen, as shown for the lowest energy site in Figure 4. The standard deviation of the energies of the site cluster about their mean is 0.03 eV.

The calculations were carried out both for addition of a positive muon and for addition of a charge neutral muonium. In both cases, a vanishingly small unpaired spin density was found at the muon site, confirming the presence of a diamagnetic muon probe state in either case. As expected, the most stable states involve addition to one of the nitrogens, breaking the Li–N bond. The resulting lowest energy structure shown in Figure 4 is almost identical between Mu addition and Mu^+^ (muon) addition. The DFT calculation provides the EFG tensor at the nitrogen site and diagonalization provides the principal values and axes and the η value. The corresponding value for *C*_*Q*_ of −2.37 MHz is derived from the principal value in both cases using the nitrogen quadrupole moment stated earlier. The η values are found to be η = 0.50 for Mu^+^ (and 0.56 for Mu). For the full cluster of sites, the average parameters were found to be *C*_*Q*_ = −2.47(5) MHz and η = 0.46(4). The predicted parameters and QLCR spectra for the calculated Mu^+^ states are consistent with the experiment (Figure 3d).

As a diamagnetic state was found for the neutral Mu addition with similar EFG parameters to that of the Mu^+^ state, this suggests that the extra unpaired electron in this case is highly delocalized within the crystal. In this particular case, one-dimensional diffusive motion of this electron is expected, leading to an intermittent hyperfine interaction with the muon. This leads to a signature *B*^–1/2^ field-dependent relaxation,^39^ which can clearly be observed in the background relaxation, as shown in the inset of Figure 2a. Although the time-averaged spin density is vanishingly small, the hyperfine interaction becomes active for the short periods of time when the spin is on the molecule containing the muon. Taking a value of 85 MHz for the hyperfine coupling in TCNQ,^19^ the diffusion rate can be estimated to be of order 3 × 10^13^ s^–1^. We note that the average transfer integral in LiTCNQ was previously estimated to be 0.06 eV,^28^ which corresponds to an electronic transfer rate of order 1.5 × 10^13^ s^–1^, showing good agreement with the μSR value.

## Discussion and Conclusions

Both the experiment and calculation indicate that in LiTCNQ, a positive muon is attracted to the N site, with the final state being diamagnetic. This particular system is a spin-Peierls insulator and remains in a spin singlet state when the muon is added. The state resulting from positive muon addition to LiTCNQ shows a well-defined QLCR, which, being sensitive to the TCNQ charge state, indicates a static charge environment at 90 K where our detailed measurements have been made. Loss of the QLCR resonances at higher temperatures may signify the onset of significant charge fluctuations, which are expected to be strongly coupled to the vibrational excitations. In contrast, Mu addition at 90 K injects an unpaired spin into the system. This is achieved by the addition of muonium to a TCNQ anion, where the negative charges (radical electrons) on the TCNQ and muonium form a singlet state or sigma bond. In the spin-Peierls state, this then leads to an unpaired electron on the TCNQ dimer resulting in a highly dynamic state, where the muon provides a local spin probe of the one-dimensional spin dynamics that is a characteristic of this type of system. From fitting the zero-field data (see Supporting Information) to the sum of a Gaussian relaxation term (representing the positive muon state) and a Risch-Kehr relaxation term^39,40^ (representing the neutral Mu state), we estimate that approximately 60% of the muons thermalize through the Mu channel and thus 40% through the positive muon channel. This work has shown that the muon can be a sensitive local probe of molecular behavior, where by use of QLCR measurements, one is able to deduce both the final stopping site of the muon and also see that the muon can provide a probe of both charge- and spin-related phenomena.

Beyond the specific LiTCNQ system studied here, a broad range of applications can be envisaged for QLCR in many other charge transfer salts containing CN or primary/secondary amine groups, covering diverse charge-related phenomena such as charge ordering, for example, κ-(BEDT-TTF)_2_Hg(SCN)_2_Cl,^41^ giant ferroelectricity, for example, κ-(BEDT-TTF)_2_Cu[N(CN)_2_]Cl,^42^ and metallic charge density wave systems, for example, TTF-TCNQ.^43^ We also note that a nitrogen QLCR transition in the region of 5 mT was previously observed in the molecular metal systems (DMe-DCNQI)_2_X (X = Cu,Li) and (DI-DCNQI)_2_Cu.^44,45^ Besides N, another common quadrupolar nucleus present in many charge transfer salts is Cl with *I* = 3/2. This nucleus has larger quadrupolar and dipolar moments compared to N, and so, it could also be a promising muon QLCR probe. Finally, turning toward a rather different direction, we note that N is universally present in the peptide structure that is the protein building block, and so, there may also be great potential for applying muon QLCR to studies of such biomolecules.

## Supporting Information Available

Properties obtained for the cluster of six lowest energy positively charged sites found using DFT for both the addition of Mu^+^ to the N site and the addition of Mu^0^ to the N site, analysis of the zero-field muon time spectra with two components, and Risch-Kehr and a static Gaussian relaxation (PDF)
