# Supplementary material for: Muon–Nitrogen Quadrupolar Level Crossing Resonance in a Charge Transfer Salt
Source: J Phys Chem C Nanomater Interfaces. 2022 Apr 26;126(17):7529–34. doi: 10.1021/acs.jpcc.2c00617 (PMC9082611; doi:10.1021/acs.jpcc.2c00617)
Supplement: Supplementary file 1 — jp2c00617_si_001.pdf [file jp2c00617_si_001.pdf]

Supplementary Information: Muon-Nitrogen Quadrupolar Level Crossing Resonance in a Charge Transfer Salt

Adam Berlie[1], Francis L. Pratt[1], Benjamin M. Huddart[2], Tom Lancaster[2] and Stephen P. Cottrell[1]

1. ISIS Neutron and Muon Source, STFC Rutherford Appleton Laboratory, Chilton, Oxfordshire, OX11 0QX, United Kingdom
2. Department of Physics, Durham University, South Road, Durham, DH1 3LE, United Kingdom

Table S1 Properties obtained for the cluster of six lowest energy positively charged sites found using DFT. These all represent the addition of Mu^+ to the N site.

Site label	E-E_0 (eV)	Mu-N distance (Å)	C-N-Mu angle (degrees)	Mu-Li distance (Å)	C_Q (MHz)	η
Pos0	0	1.031	127.7	2.58	-2.37	0.501
Pos1	0.042	1.038	120.9	2.62	-2.47	0.511
Pos2	0.054	1.037	121.0	2.56	-2.49	0.406
Pos3	0.066	1.041	116.8	2.85	-2.54	0.428
Pos4	0.081	1.037	119.9	2.82	-2.47	0.463
Pos5	0.092	1.041	122.2	2.53	-2.46	0.459

Table S2 Properties obtained for the cluster of six lowest energy charge-neutral sites found using DFT. These all represent the addition of Mu^0 to the N site.

Site label	E-E_0 (eV)	Mu-N distance (Å)	C-N-Mu angle (degrees)	Mu-Li distance (Å)	C_Q (MHz)	η
Mu0	0	1.037	127.9	2.51	-2.37	0.563
Mu1	0.074	1.032	125.2	2.55	-2.46	0.454
Mu2	0.102	1.040	119.9	2.59	-2.47	0.547
Mu3	0.102	1.037	119.9	2.49	-2.52	0.418
Mu4	0.118	1.041	116.5	2.82	-2.50	0.456
Mu5	0.120	1.037	119.3	2.64	-2.56	0.429

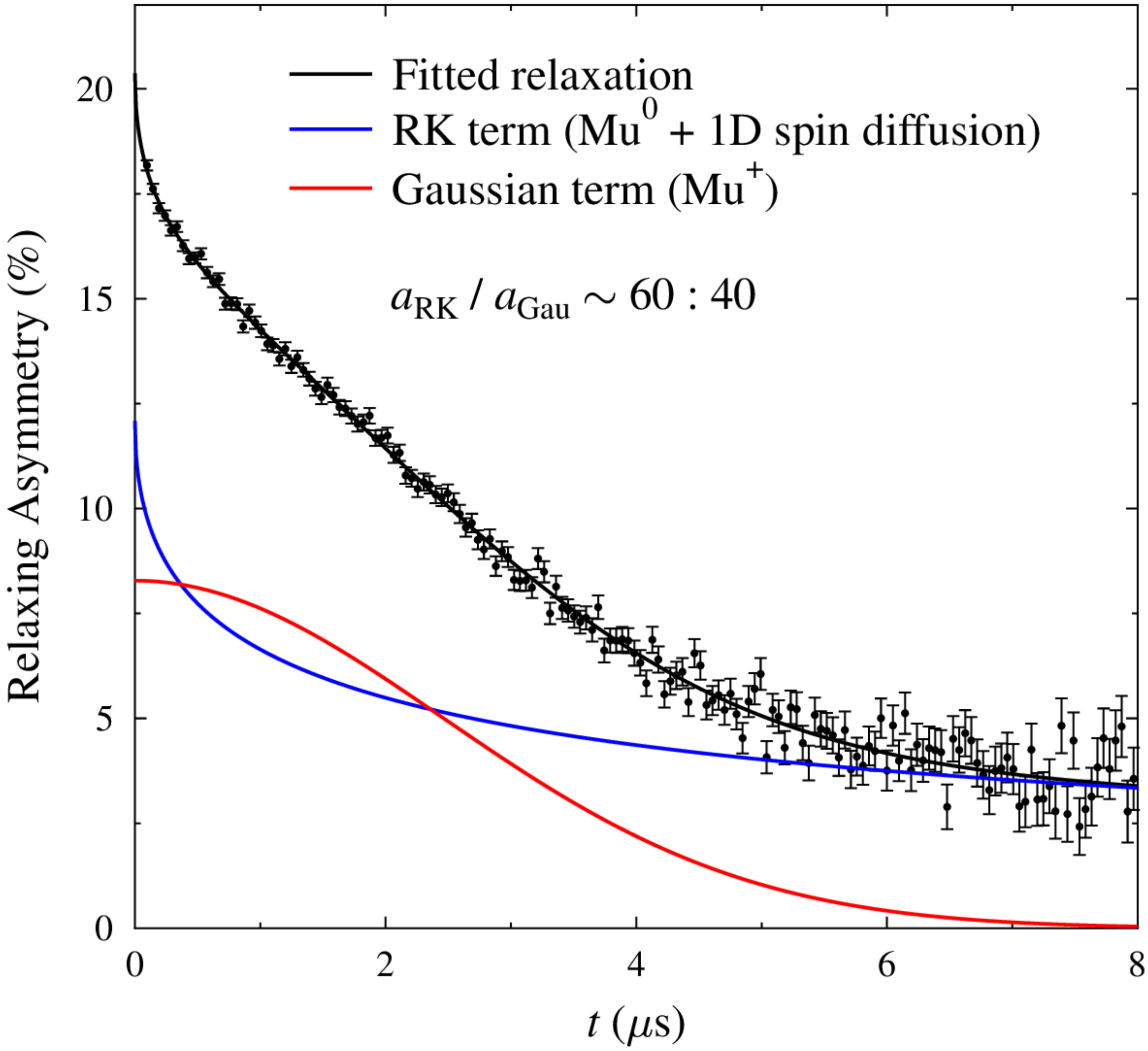

Figure S1 Resolution of the relaxation function at zero field into two components. The Risch-Kehr (RK) term represents the relaxation from 1D spin diffusion, which follows on from Mu^0 addition to the N site. The Gaussian term represents the diamagnetic state resulting from Mu^+ addition to the N site and this is the component responsible for the nitrogen QLCR resonances observed at specific longitudinal fields. The amplitude ratio of the two terms is of order 60 to 40.
